# A TECHNOLOGY-BASED TRANSITIONAL PALLIATIVE CARE INTERVENTION FOR FAMILY CAREGIVERS IN RURAL OR UNDERSERVED AREAS

**DOI:** 10.1093/geroni/igae098.2111

**Published:** 2024-12-31

**Authors:** Joan Griffin, Diane Holland, Catherine Vanderboom, Jay Mandrekar, Brystana Kaufman, Allison Gustavson, Cory Ingram

**Affiliations:** Mayo Clinic, Rochester, Minnesota, United States; Mayo Clinic, Rochester, Minnesota, United States; Mayo Clinic, Rochester, Minnesota, United States; Mayo Clinic, Rochester, Minnesota, United States; Duke University School of Medicine, Durham, North Carolina, United States; Minneapolis Veterans Affairs Health Care System, Minneapolis, Minnesota, United States; Mayo Clinic, Rochester, Minnesota, United States

## Abstract

In this presentation, Dr. Griffin will describe the Technology-Enhanced Transitional Palliative Care (TPC) study, a randomized controlled trial testing the efficacy and cost-effectiveness of an intervention to address FCGs’ unmet needs in order to improve their health and well-being. Nurse interventionists use video visits to teach, guide and counsel FCGs in rural areas caring for someone receiving palliative care and transitioning form hospital to home. In addition to providing background on the importance of addressing unmet family caregiver needs during care transitions for patients receiving palliative care, she will describe the study design, sample, intervention and control group procedures, including the use of video visits, and outcomes assessed. This presentation will provide necessary context for the remaining presentations.

